# Global Epidemiology of *Plasmodium vivax*

**DOI:** 10.4269/ajtmh.16-0141

**Published:** 2016-12-28

**Authors:** Rosalind E. Howes, Katherine E. Battle, Kamini N. Mendis, David L. Smith, Richard E. Cibulskis, J. Kevin Baird, Simon I. Hay

**Affiliations:** 1 Spatial Ecology and Epidemiology Group, Department of Zoology, University of Oxford, Oxford, United Kingdom.; 2 Center for Global Health and Diseases, Case Western Reserve University, Cleveland, Ohio.; 3 Global Malaria Program, World Health Organization, Geneva, Switzerland.; 4 Fogarty International Center, National Institutes of Health, Bethesda, Maryland.; 5 Sanaria Institute for Global Health and Tropical Medicine, Rockville, Maryland.; 6 Institute for Health Metrics and Evaluation, University of Washington, Seattle, Washington.; 7 Eijkman-Oxford Clinical Research Unit, Jakarta, Indonesia.; 8 Centre for Tropical Medicine and Global Health, Nuffield Department of Medicine, University of Oxford, Oxford, United Kingdom.; 9 Wellcome Trust Centre for Human Genetics, University of Oxford, Roosevelt Drive, Oxford, United Kingdom.

## Abstract

*Plasmodium vivax* is the most widespread human malaria, putting 2.5 billion people at risk of infection. Its unique biological and epidemiological characteristics pose challenges to control strategies that have been principally targeted against *Plasmodium falciparum*. Unlike *P. falciparum*, *P. vivax* infections have typically low blood-stage parasitemia with gametocytes emerging before illness manifests, and dormant liver stages causing relapses. These traits affect both its geographic distribution and transmission patterns. Asymptomatic infections, high-risk groups, and resulting case burdens are described in this review. Despite relatively low prevalence measurements and parasitemia levels, along with high proportions of asymptomatic cases, this parasite is not benign. *Plasmodium vivax* can be associated with severe and even fatal illness. Spreading resistance to chloroquine against the acute attack, and the operational inadequacy of primaquine against the multiple attacks of relapse, exacerbates the risk of poor outcomes among the tens of millions suffering from infection each year. Without strategies accounting for these *P. vivax*-specific characteristics, progress toward elimination of endemic malaria transmission will be substantially impeded.

## Introduction

*Plasmodium vivax* and *Plasmodium falciparum* are the primary causes of malaria in humans and until recent years, the majority of malaria research and funding has been focused on the prevention, treatment, and control of *P. falciparum*.^1^ Both parasite species expose approximately 2.5 billion people to risk of infection.^2^,^3^ Although *P. falciparum* causes many deaths, especially in sub-Saharan Africa where *P. vivax* is rarer, the predominance of *P. vivax* in some of the world's most densely populated and impoverished regions, coupled with its now proven association with severe and fatal outcomes, informs the importance of reversing the historic neglect of this infection. A deeper understanding of the unique epidemiology of this parasite is necessary for effective malaria control and elimination efforts. A synthesis of the current status of this knowledge represents this review's objective.

Important variances in the biology of *P. vivax* and *P. falciparum* render their epidemiologies distinctly different. The most apparent is the ability of *P. vivax* to cause relapses weeks to months following a primary infection by activation of dormant liver-stage parasites, known as hypnozoites. These dormant liver-stage infections constitute what is called the hypnozoite reservoir of infection. That reservoir streams new blood infections and clinical attacks into these communities, along with opportunities for onward transmission. The hypnozoite reservoir extends clinical attacks across seasons inhospitable to anopheline mosquitoes, effectively widening its natural geographic range far into temperate zones including the Korean Peninsula.^2^

There also exist differences in the blood-stage dynamics that impact *P. vivax* epidemiology. Natural immunity is acquired at a younger age than against *P. falciparum*, making infants the primary risk group for severe vivax malaria in heavily endemic settings.^4^ In low-transmission settings, where *P. vivax* often persists against elimination efforts, all age groups appear at risk of severe disease.^4^ The propensity for *P. vivax* to invade young blood cells (reticulocytes) and its ability to migrate beyond the venous sinuses results in relatively low levels of parasitemia.^5^ Even in low-transmission settings, most infections appear to be microscopically sub-patent and asymptomatic.^4^

Low blood-stage parasitemia does not make *P. vivax* a “benign” infection, as once believed.^5^–^7^ Despite low levels of detectable parasites, *P. vivax* causes significant morbidity and can be associated with severe malaria and death.^5^,^8^–^20^ Remarkably, the primary therapies against such outcomes, chloroquine and primaquine, have been in continuous use since 1952, despite worsening resistance to chloroquine and the operational inadequacy of primaquine where most malaria patients live and seek treatment.

This article reviews the epidemiology of *P. vivax* in the context of these crucial issues. The geographic distribution of *P. vivax* infection and differences in relapse phenotype are illustrated and described alongside estimates of *P. vivax* burden, as well as severe, lethal, and chloroquine-resistant vivax malaria to demonstrate that control and elimination strategies developed for *P. falciparum* cannot simply be transferred to *P. vivax*.^21^–^25^ The problems imposed by the unique biology of *P. vivax* require the development and implementation of a control and elimination strategy enabled by a relatively modest new set of tools.^26^

## Geography of *P. vivax* Infection

*Plasmodium vivax* occurs across the widest geographic area of the human malarias, extending well beyond the limits of *P. falciparum* into temperate climates. This is enabled by intracellular parasites dormant in the human liver, a safe haven from immune attack during long mosquito-free cold seasons when onward transmission and propagation is not possible.

*Plasmodium vivax* depends on the Duffy antigen to invade red blood cells. Individuals who do not express the Duffy antigen have, therefore, been considered refractory to *P. vivax* infection^27^ and the prevalence of Duffy negative populations must be considered in predictions and maps of *P. vivax* endemicity. *Plasmodium vivax* was long thought to be absent from parts of Africa where the Duffy negative phenotype occurs at very high frequencies.^28^ Recent findings of *P. vivax* infections in Duffy negative patients raise the possibility of an alternative invasion mechanism to Duffy, but further investigation is required to assess the public health significance of these findings.^29^,^30^ The global map of *P. vivax* presented here takes into account estimates of Duffy negativity prevalence as a proxy of the population refractory to infection.^28^ The regular occurrence of *P. vivax* diagnoses in European and American patients who traveled only to areas of Africa dominated by Duffy negativity,^31^ and a formal review of the evidence of the *P. vivax* transmission in Africa,^32^ emphasizes the importance of resisting the temptation to regard these zones as free of *P. vivax* risk.

**Figure 1.**
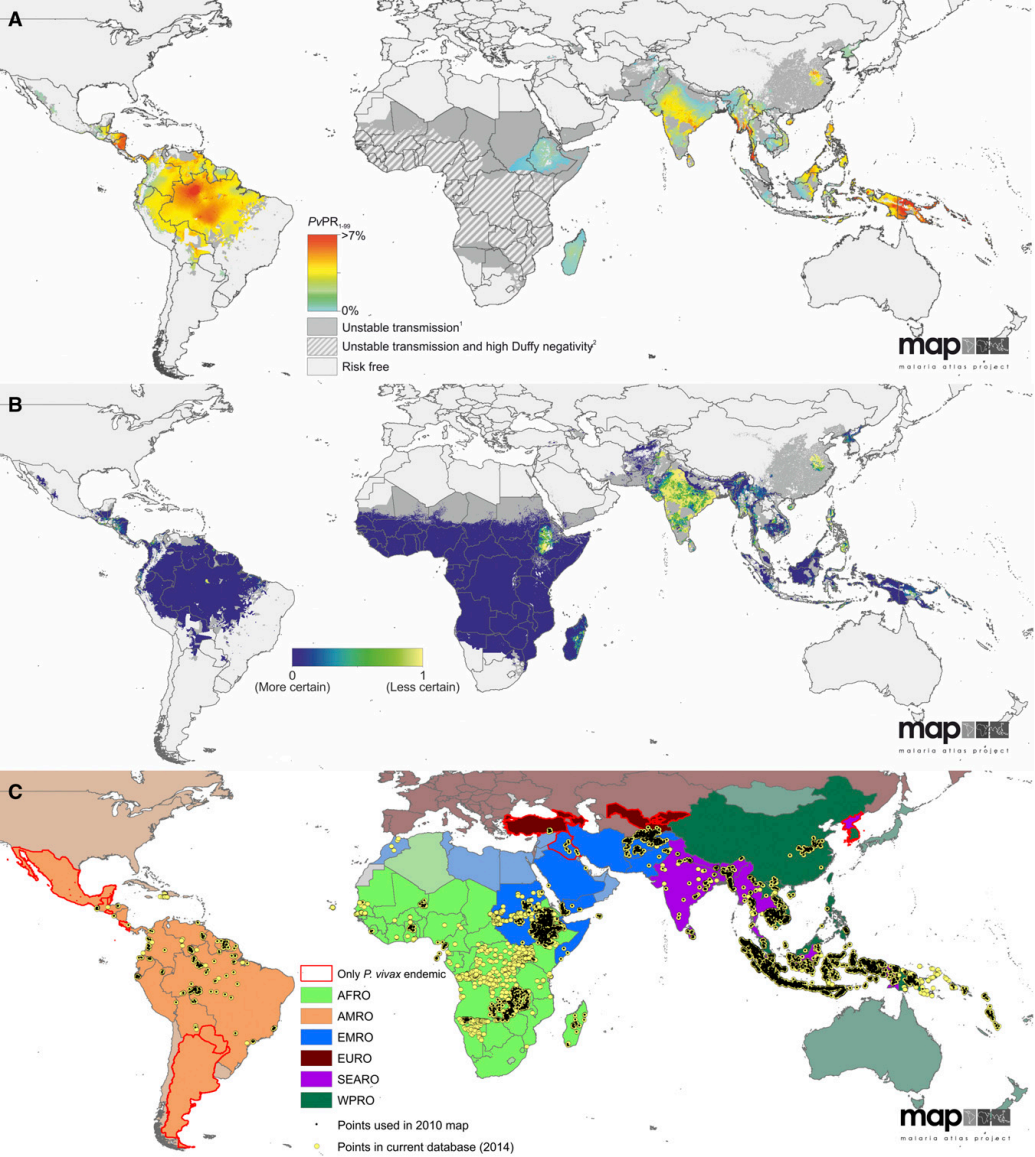
The spatial distribution of *Plasmodium vivax*, associated uncertainty, and input data records. (**A**) The limits and endemicity of *P. vivax* in 2010.^2^ Spatial limits of parasite-specific malaria risk are defined by annual parasite incidence (*Pv*API) with further medical intelligence, temperature, and aridity masks. Areas were defined as stable, unstable (dark grey areas, *Pv*API < 0.1 per 1,000 per annum), or no risk (light grey). The model-based geostatistics point estimates of the annual mean predicted prevalence are shown within the spatial limits of stable transmission. Estimates of parasite rate standardized to 1- to 99-year-olds (*Pv*PR_1–99_) that range from 0% to > 7% are shown as a spectrum of blue to red. Hatching indicates areas where Duffy negativity gene frequency is predicted to exceed 90%.^28^ (**B**) The population-weighted uncertainty as the ratio of the posterior interquartile range to the posterior mean prediction at each pixel on a blue to yellow color spectrum multiplied by the underlying population density and rescaled to 0–1. Higher values (yellow) indicate areas with high uncertainty and large populations. (**C**) World Health Organization regions by color: the African region (AFRO) in green, the region of the Americas (AMRO) in orange, the eastern Mediterranean region (EMRO) in blue, the European region (EURO) in burgundy, the southeast Asian region (SEARO) in purple, and the western Pacific region (WPRO) in dark green. The countries in each region that are not endemic for *P. vivax* are slightly greyed out and shaded a lighter color. Those countries that are endemic only with *P. vivax* malaria are outlined in red. The location of the prevalence surveys that were input into the model that produced the map in Figure 1A is shown as small black points and the surveys conducted since 2010 are shown in yellow, illustrating the increased attention given to *P. vivax* in recent years.

Modeled spatial data of *P. vivax* transmission and prevalence,^2^,^31^ the uncertainty of those estimates, and the current data available to inform those predictions are presented as maps in Figure 1

. The methods used to generate these figures are described in detail elsewhere.^33^ Table 1 shows estimates of the population at risk (PAR) of infection, calculated by combining the maps of the limits of transmission with gridded population surfaces.^34^,^35^

Levels of *P. vivax* endemicity vary widely among the World Health Organization (WHO) regions. Outside of Africa, *P. vivax* is the dominant species, with relatively high prevalence of infection in the South-East Asian and Western Pacific regions.^36^ Relatively high endemicity also occurs in most of the Americas, but in much less densely populated areas. Data from which to estimate the geographic extent of *P. vivax* transmission in Africa are limited.^32^ High *P. falciparum* endemicity in much of that continent coupled with high prevalence of Duffy negativity overshadowed collection of *P. vivax*-specific data as a priority.^25^ Indirect evidence from returning travelers suggests that *P. vivax* is present at low endemicity in almost all sub-Saharan African countries.^31^,^32^

How *P. vivax* transmission may be sustained in populations known to be predominantly Duffy negative is not well understood. However, in the instance of *Plasmodium ovale* in the Asia Pacific, such chronic endemicity without measurable prevalence is known.^37^ The biology of *P. vivax*, in particular its ability for repeated relapses from a single mosquito inoculation, coupled with the very early emergence of gametocytes in the course of blood-stage infection, perhaps enables parasite survival despite relatively low probability of propagation in blood. Further, population mobility along with immigration may amplify the heterogeneity of the Duffy phenotype and increase the population of vulnerable Duffy-positive hosts able to sustain infection.

Some of the highest predicted *P. vivax* prevalence estimates in 2010 (Figure 1) have a relatively sparse evidence base of survey data for large parts of the world (e.g., Brazil, India, Myanmar, and Papua New Guinea). Twenty of the 95 *P. vivax* malaria–endemic countries are in the southeast Asia and western Pacific regions, and their populations comprise 83% of the global PAR of *P. vivax*.

**Figure 2.**
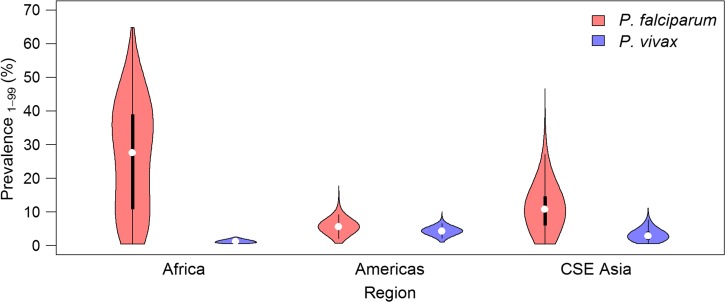
Comparison of *Plasmodium falciparum* and *Plasmodium vivax* prevalence.^135^ Prevalence values from *P. falciparum* and *P. vivax* endemicity surfaces^2^,^3^ standardized to the 1- to 99-year age range.^72^ The shaded areas correspond to each species and show a smoothed approximation of the frequency distribution (a kernel density plot) of parasite prevalence within each geographic region. The black central bar represents the interquartile range and the white circles indicate the median values. CSE Asia = Central and Southeast Asia.

**Figure 3.**
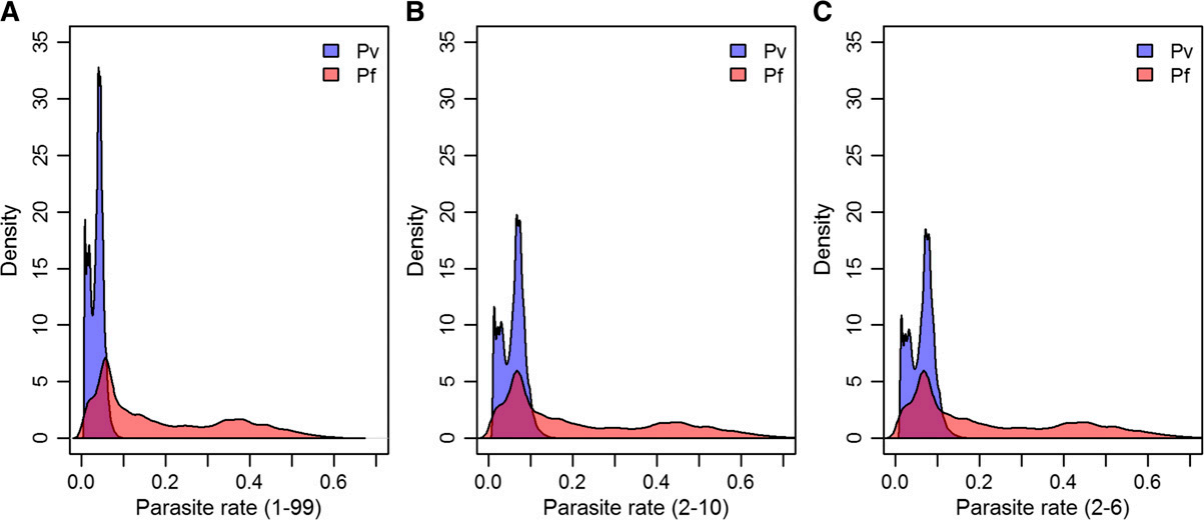
Density plots of parasite rate (PR) pixels for *Plasmpdium falciparum* and *Plasmodium vivax* in all regions excluding Africa. The plot (**A**) shows the PR values age standardized^72^,^191^ to all ages (1–99 years), (**B**) is standardized to 2- to 10-year-olds, and (**C**) 2- to 6-year-olds. The plots show that regardless of age, the vast majority of *P. vivax* is found at lower prevalence values.

The prevalence of *P. vivax* predicted within 5 km^2^ areas globally (Figure 1) was derived from a sample of 9,970 parasite rate (PR) surveys.^2^ Compared with similar predictions for *P. falciparum*,^3^ relatively low prevalence occurs throughout the endemic world. The central tendency of the *P. vivax* PR (*Pv*PR) estimates, age standardized to the 1- to 99-year age range (*Pv*PR_1–99_), rarely exceeded 10%.^2^ As shown in Figure 2

, the prevalence of *P. vivax* is universally low compared with *P. falciparum*, both in range and median values. The 1–99 age range was applied to *P. vivax*, rather than the 2–10 years range used for *P. falciparum*,^3^ because *P. vivax* cross-sectional surveys were most often conducted in whole populations.^2^ The patterns of *Pv*PR and *P. falciparum* PR (*Pf*PR) by age also differ slightly. *Plasmodium vivax* prevalence peaks in children aged 2–6, compared with 2–10 for *P. falciparum*. In adults, *Pv*PR is also lower than *Pf*PR when considered as a proportion of the peak: *Pv*PR in adults is roughly a quarter of *Pv*PR_2–6_ whereas *Pf*PR in adults is roughly a third of *Pf*PR_2–10_. PR for either parasite is lower in the whole population than in children, but a direct comparison of PR in children relative to whole populations illustrates that *P. vivax* prevalence tends to be much lower regardless of age group (Figure 3

).

There are other reasons why *Pv*PR could be lower than *Pf*PR. Although the prevalence of *P. vivax* in heavily endemic areas may reach or even exceed that of *P. falciparum*, *P. vivax* can often go undetected.^38^ Low parasite densities lead to high rates of false-negative diagnoses by microscopy or rapid diagnostic tests (RDTs).^39^ Microscopy diagnoses underestimate the true prevalence of blood-stage *P. vivax* in both high- and low-transmission settings.^40^–^47^ *Plasmodium vivax* in mixed infections is also often underdiagnosed^48^; lower densities of *P. vivax* in peripheral blood translate into *P. falciparum* being diagnosed before *P. vivax* is spotted by the microscopist.

Another consideration with regard to true versus observed prevalence is the invisibility of the dormant liver stages to any diagnosis. These parasites could be highly prevalent in many endemic settings. A report by Douglas and others^49^ from Thailand offers some evidence of this. Two months after antimalarial treatment of blood-stage *P. falciparum* infection (which would also have been active against blood-stage *P. vivax* parasites), 51% of patients tested positive for blood-stage *P. vivax*. Given the region's relatively low transmission rate, this suggests a high proportion of hypnozoite-derived infections and provides an estimate of the prevalence of latent *P. vivax* in people having acute falciparum malaria. Further, *P. vivax* may sequester in tissues like bone marrow and the hemopoietic regions of the spleen.^5^ In at least one patient with asymptomatic and microscopically sub-patent parasitemia, the spleen was heavily laden with blood-stage trophozoites of *P. vivax*.^50^

All of these findings recommend caution in comparing prevalence estimates of *P. vivax* and *P. falciparum* based largely on blood-stage infections diagnosed by relatively insensitive means. These may underestimate true prevalence of *P. vivax* far more than *P. falciparum*. The limitations of diagnostics technologies to detect actual parasite density and actual parasite mass in any given host (or population) have been compared with the floating and submerged sections of an iceberg. In this analogy, one may reasonably assign a greater specific gravity to the *P. vivax* iceberg—it is likely that much more of it is submerged (by intrinsically low blood-stage infection and diagnostically invisible parasites sequestered in marrow and spleen or lying dormant in the liver) relative to *P. falciparum*.

## Relapse Epidemiology

**Figure 4.**
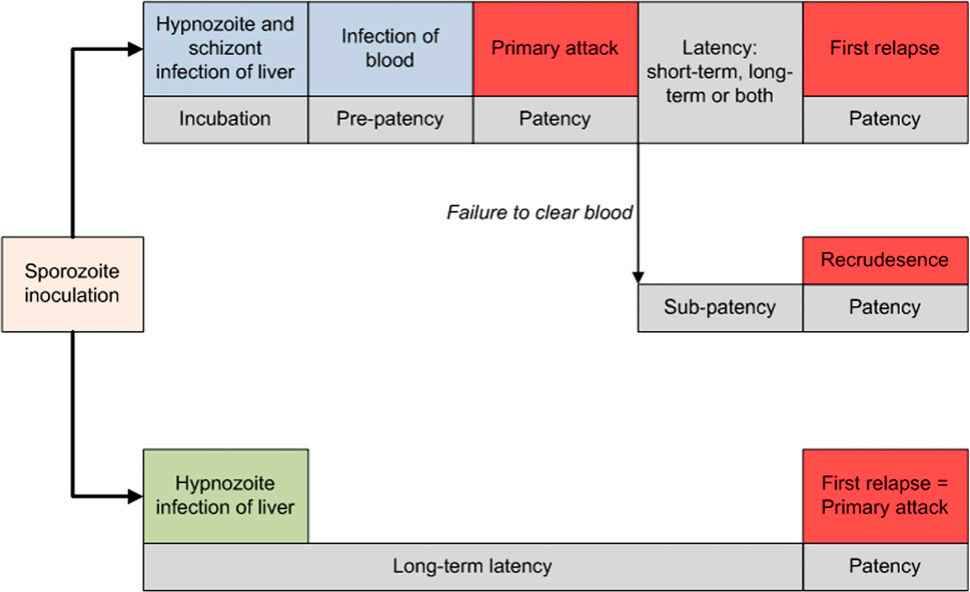
Pathways to infection of blood and clinical attacks in *Plasmodium vivax* malaria.^51^

The hypnozoites cause multiple clinical attacks from a single bite of a *P. vivax*-infected mosquito. In contrast, *P. falciparum* infection of the liver yields a single blood-stage infection. In other words, “infection” as an event in *P. falciparum* is singular and clear, whereas the same in *P. vivax* takes on complex plurality and ambiguity in an epidemiological sense (Figure 4

).^51^,^52^

Recurrence of asexual *P. vivax* parasites in peripheral blood may derive from three distinct sources: relapse (from hypnozoites), recrudescence (from sub-patent asexual parasitemia), or reinfection (by new mosquito inoculation of sporozoites).^53^ Assigning the source of any given parasitemia observed in endemic settings is virtually impossible.^54^,^55^ Nonetheless, it may be inferred at a population level: the force of infection attributable to sporozoites versus hypnozoites can be estimated through randomized drug trials comparing recurrence rates between treatment arms with and without primaquine. Such investigation in a highly endemic area of Papua New Guinea, where parasites have a short relapse frequency of about a month,^51^ estimated that relapses caused approximately 50% of blood-stage infections and more than 60% of clinical episodes in the first 3 months following the drug intervention.^56^–^58^ Relapse may well be the predominant origin of most *P. vivax* clinical attacks throughout the endemic world.

Geographical variation in the rate and timing at which a “strain” of *P. vivax* may relapse has long been known.^59^–^61^ Temperate and subtropical strains of the parasite exhibit either a long incubation period or a long delay between the primary infection and relapse (around 8–10 months). Tropical strains are characterized by short incubation times and short relapse intervals.^62^ How hypnozoite relapses are triggered, and the source of this phenotypic variation, remain unknown.^39^ The relapse mechanism may be an adaptive trait of the parasite to sequester or “hibernate” during conditions inhospitable to the *Anopheles* vectors.^63^–^65^ Another theory is that latent hypnozoites are activated by systemic febrile illness,^65^,^66^ which explains observations of multiple relapses at regular (seemingly triggered) intervals, heterologous genotypes in relapse, and what seems to be a high frequency of relapses following *P. falciparum* infections.^49^,^65^,^67^ Others hypothesized that a mosquito bite (and its complex immunologic consequences) may trigger relapse.^68^ However, observations from sporozoite-induced infections in North American prisoner volunteers during the 1940s and 1950s indicate relapses occur at predictable intervals and rates, without any known trigger or stimulus.^51^ Further, findings of Shute and others^64^ suggested that individual sporozoites were genetically programmed for either direct tissue development with early primary attack, or for latency and very late primary attack. They observed early primary attacks with the typically long-term latent North Korean strain, but only when the sporozoite inoculum was very high: this strain had relatively high frequencies of sporozoites with genetically set latency, and those set for rapid development were so rare as to not ordinarily occur with the normally low numbers of sporozoites of natural inoculation.

**Figure 5.**
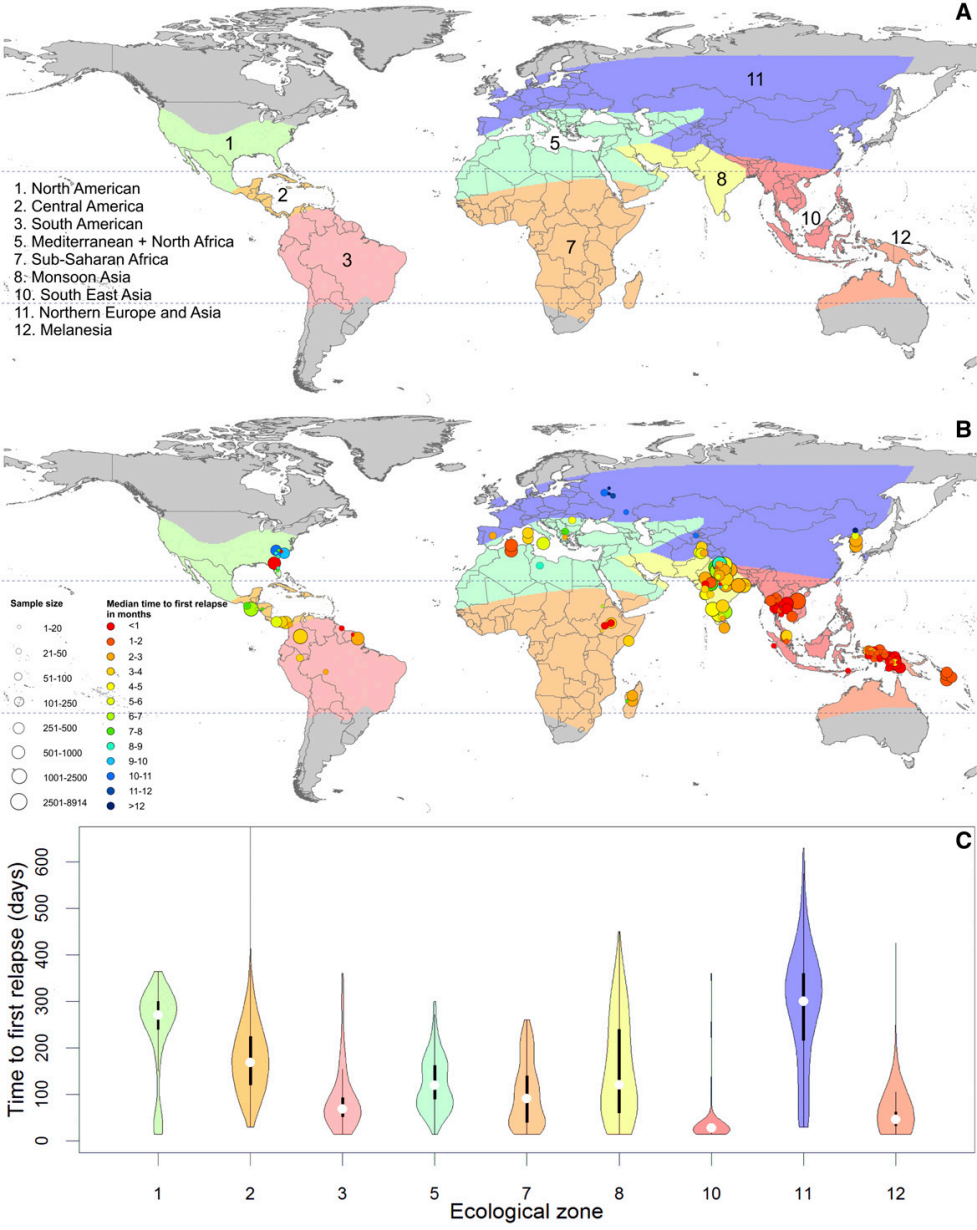
Zoo-geographical zones and observed time to first relapse.^51^ (**A**) The zoo-geographical zones used to describe the time to first relapse. (**B**) The median observed time to relapse in each study used to obtain individual data. The size of each point varies by sample size and the time to first relapse is shown on a spectrum of red (< 1 month) to dark blue (> 12 months). (**C**) Violin plots show the observed time to first relapse in individuals from each zone in Figure 5A. The colored areas correspond to each zone and show a smoothed approximation of the frequency distribution (a kernel density plot) of the time to relapse within each geographic region. The black central bars represent the interquartile range, and the white circles indicate the median values.

**Figure 6.**
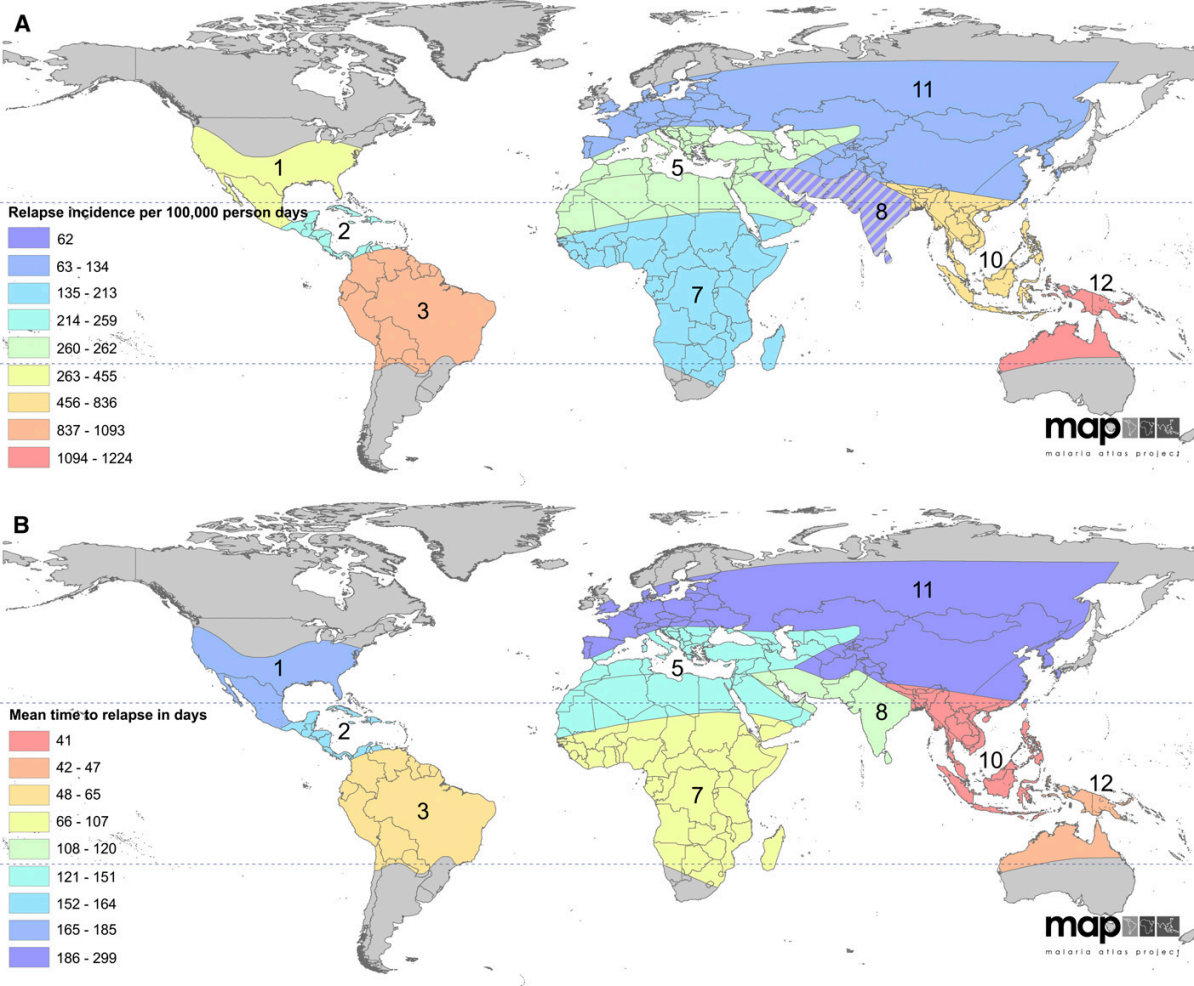
Modeled relapse incidence and mean time to relapse.^51^ (**A**) The relapse incidence per 100,000 person days on a spectrum of blue to red, with red being the highest incidence of relapse. Zone 8 is hatched to indicate that the prediction is to be interpreted with caution. (**B**) The predicted mean time to relapse on a spectrum from blue to red, with red being most frequent relapse. The numbers of the zones correspond to those shown in Figure 5A.

Evidence from records of relapse observed in both controlled experimental and natural settings has demonstrated geographical variation in the timing of relapse. A meta-analysis of over 5,000 first relapse events revealed that epidemiological zones outlined by Macdonald^69^ describe the observed heterogeneity in relapse.^51^ The zones, map, and violin plots in Figure 5

illustrate the variation in time from primary infection to first relapse observed in each area. Although a wide range was observed in almost every zone, some regions had a clear tendency to short or long times to relapse (Figure 6

).

Regionally specific relapse patterns should be considered when assessing control and elimination strategy and tactics. The impact of vector control on the incidence and prevalence of *P. vivax* malaria may be less rapid than that observed for *P. falciparum,* with the magnitude of the difference dependent on the relapse phenotype of the region.^65^ Effects may be slowest to materialize in regions characterized by long-latency strains or high rates of relapse (even in areas with shorter latency). The Americas, South-East Asia, and Western Pacific WHO regions all harbor strains that relapse quickly and repeatedly following primary infection. The Indian subcontinent and sub-Saharan Africa have variable relapse frequencies and periodicity, but show moderate time to relapse overall. Longer times to relapse are shown in areas around the Mediterranean and Central America, with the longest periods of latency in China and the Korean Peninsula. However, the presence of long-latency strains may be widespread. It is difficult to exclude their presence among multiple frequently relapsing strains in the tropics.^65^ These cautions, coupled with the fears of potential primaquine-induced hemolysis that sharply impede its effectiveness, highlight the complexity and difficulty that relapse brings to *P. vivax* epidemiology and control.^70^

## High-Risk Groups

The population groups at highest risk of *P. vivax* infection are determined by immunological factors directly associated with intensity of local transmission, host genetics, and behavioral traits affecting exposure to infectious bites. As such, the primary risk groups differ between epidemiological settings and shift as endemicity wanes toward elimination.^71^ Key risk factors are considered here, though comorbidities and malnutrition, which are discussed later in relation to the clinical aspects of severe disease, are also important risk factors for infection and severe outcomes.

### Age.

**Figure 7.**
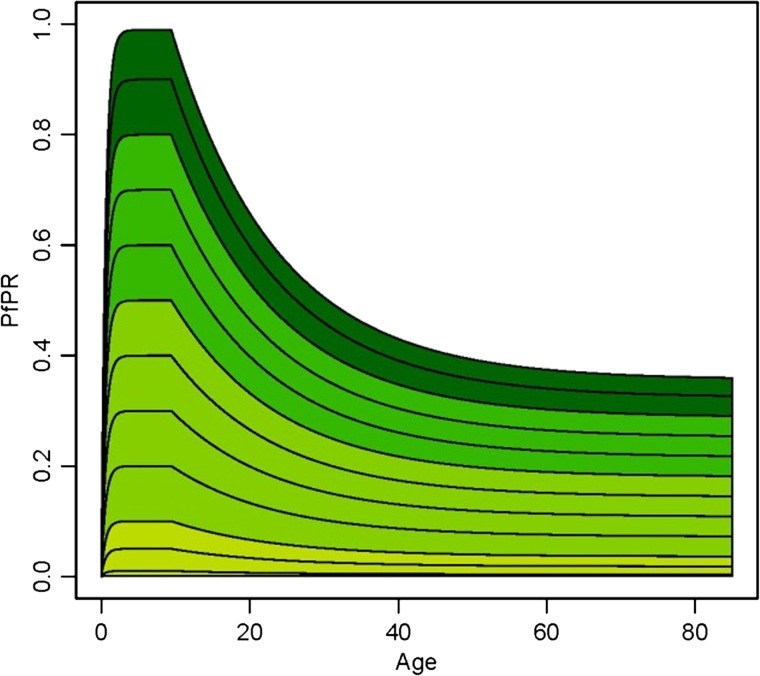
Schematic of the age–parasite rate relationship by endemicity class. The curves generated by a model for *Plasmodium falciparum* (*Plasmodium vivax* would follow a similar pattern) show the age–parasite relationship at different endemicity levels: holoendemic areas are dark green (category of highest transmission levels), hyperendemicity areas green, mesoendemic areas light green, and hypoendemic areas olive green. Figure reproduced from Smith and others (2007).^72^

The distribution of *Plasmodium* infections across a community varies in a predictable pattern as a function of age and transmission intensity.^2^,^4^,^72^ In relatively higher endemic areas, young children bear the brunt of disease burden, because by adolescence individuals will have developed immunity against symptomatic disease. In the far more common low-transmission settings, however, infections may not occur frequently enough to allow protective immunity to develop, and in these communities, symptomatic infection is more evenly distributed across age groups.^4^ Figure 7

characterizes these patterns in different endemicity settings. The pattern applies to both *P. falciparum* and *P. vivax*, but studies from co-endemic high-transmission areas of Papua New Guinea and eastern Indonesia report an earlier peak in *P. vivax* cases around 2 years of age compared with 5–10 years for *P. falciparum*, reflecting “faster” acquisition of immunity to *P. vivax* (the role of age per se versus cumulative exposure in these rates is unclear).^12^,^43^,^73^–^78^ In these high-endemicity settings, clinical *P. vivax* is relatively rare in children older than 5 years. This earlier age peak for *P. vivax* has also been reported from lower transmission intensity settings in Sri Lanka,^79^ Thailand^80^, and Myanmar.^81^ Reasons for this disparity between rates of natural acquisition of immunity against both parasites remain uncertain,^4^ but studies of laboratory-induced infections during the 1930s suggested that acquisition of immunity with fewer clinical attacks was an intrinsic property of *P. vivax*.^82^ Alternatively, it has also been hypothesized to result from greater exposure to *P. vivax* blood-stage parasitemia due to relapses.^11^,^77^ Case data from areas nearing elimination (such as Sri Lanka and Malaysia)^71^ also reflect these age shifts in disease burden from young children to all ages as a function of the changing transmission intensity over time.^78^

### Ecological setting.

Residents of urban areas are generally at lower risk of malaria infection due to man-made ecological conditions that are inhospitable to most *Anopheles* vector species.^2^,^33^,^83^ *Anopheles stephensi*, common across the Arabian Peninsula and the Indian subcontinent, and east into southern China, is an exception to most malaria vectors in being well adapted to survival in urban habitats.^84^ Evidence discussed in this supplement's review of *P. vivax* epidemiology in India^85^ suggests a trend toward increasing infection prevalence in Indian cities, perhaps tied to land-use changes as part of urbanization construction projects creating breeding habitats and potentially also resulting from greater population movement between urban and rural environments. In addition, the bionomics of the *Anopheles* mosquitoes in many *P. vivax* endemic areas differ from those within Africa.^84^,^86^,^87^ For example, the exophilic and zoophilic tendencies of dominant vector species in Asia render standard malaria control strategies of indoor residual spraying and insecticide-treated nets less effective.^84^,^88^

Migrant workers represent a risk group of particular priority for malaria control efforts. Many migrants work in high-risk environments such as natural forest, palm oil or rubber plantations, and fish farms.^89^ In areas where major epidemiological shifts toward elimination have occurred, these migrant workers, predominantly adult males, sustain transmission.^71^ Their mobile behavior, together with a range of social, legal, economic, and geographic factors, limits their access to and contact with health-care delivery systems,^90^ putting themselves and those in the areas they live and transit through at sustained risk of transmission.

### Pregnancy.

Pregnant women and infants are a primary risk group for threatening clinical vivax malaria,^91^,^92^ resulting in relatively high maternal, infant, and fetal morbidity and mortality.^93^–^95^ Maternal anemia^96^ increases the risks of premature labor, stillbirth, and reduced birth weight.^93^,^97^ Studies in Thailand have found severe malaria to be three times (95% confidence interval [CI]: 1.4–6.2) more common in pregnant than nonpregnant women,^98^ and the odds of miscarriage about three times higher for women infected with either *P. falciparum* (adjusted odds ratio [AOR]: 2.7, 95% CI: 2.1–3.4) or *P. vivax* (AOR: 3.1, 95% CI: 2.4–3.9) than for women with no malaria.^99^ Data from Indonesia suggested that the trigger of severe outcomes was the symptoms of infection rather than the parasitemia per se, and prompt treatment of infections during the first trimester can prevent significant birth weight reductions.^96^ The contraindication of primaquine treatment of pregnant/lactating women and infants exposes these highly vulnerable groups to the threat inherent in repeated clinical attacks at short intervals.^100^

### Host genetic factors.

A number of seemingly deleterious human genetic traits affecting red blood cells are found with the highest frequencies in populations across malaria-endemic zones.^101^,^102^ Given this observation, the “malaria hypothesis” suggests these blood disorders may confer protection against malaria disease.^103^ Although most research has focused on *P. falciparum* (especially with sickle-cell disease), some polymorphisms have been found to confer protection against *P. vivax*. For instance, southeast Asian ovalocytosis in Papua New Guinea may confer partial resistance to infection,^104^,^105^ as may certain variants of glucose-6-phosphate dehydrogenase (G6PD) deficiency.^70^,^106^,^107^ In contrast, both alpha and beta thalassemia have been associated with increased risk of *P. vivax* parasitemia.^108^,^109^ Host genetic factors may, therefore, play a role in determining groups at increased risk of *P. vivax* infection, or groups innately protected from clinical infection, but more *P. vivax*-specific evidence is required to draw concrete conclusions and quantify the altered risk levels.

## Asymptomatic Blood-Stage Infections

Asymptomatic blood-stage *P. vivax* infections in populations are often cited as a barrier to control, much like the undetectable reservoir of hypnozoite infections in the liver. This section addresses asymptomatic blood-stage infections, whether arising from sporozoites, hypnozoites, or insufficiently treated primary infections. Infection detectability varies over the time course of *P. vivax* infections, with fever and symptoms usually occurring early, when they occur at all. Depending on the setting, typically a small proportion of blood-stage infections trigger symptoms and treatment seeking, whereas a much larger proportion of infections will be asymptomatic, sub-patent, or dormant.^41^ For infections that are not treated or improperly treated, the resolution of symptoms is followed by a much longer asymptomatic infection that is progressively less likely to be detected as it ages. Different proportions of asymptomatic and sub-patent infections will be detected based on the survey method (i.e., longitudinal versus cross sectional), the diagnostics used, and the timing of the survey with respect to recent outbreaks or transmission seasonality.

**Figure 8.**
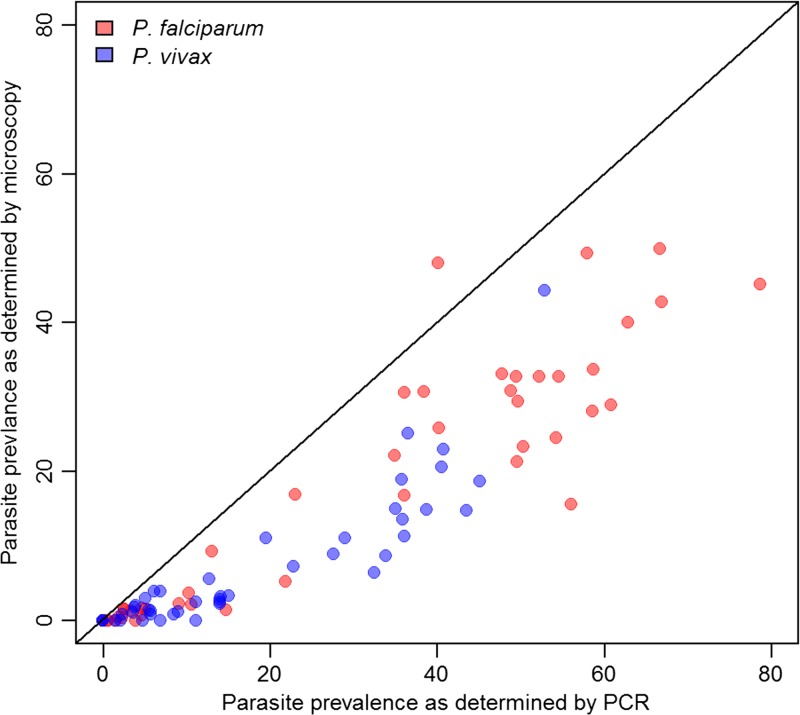
The proportion of infections detected by microscopy versus proportion detected by polymerase chain reactions (PCR) for *Plasmodium falciparum* and *Plasmodium vivax*. Derived from data in Okell and others 2012 supplementary information.^192^ Only surveys where both *P. vivax* and *P. falciparum* were detected are shown. Of the 44 data points for each species, all but four were for all age groups—the remaining four considered children under age 5 only.

It is not known what proportion of *P. vivax* infections are or will become febrile or otherwise symptomatic, but the majority of *P. vivax* infections in cross-sectional surveys are symptomless and undetectable by standard diagnostic techniques (RDTs and microscopy).^4^ Up to 97% of microscopically diagnosed *P. vivax* infections in the Pacific Asia region have been reported to be asymptomatic; higher sensitivity diagnostics, such as polymerase chain reaction (PCR), would likely find an even higher proportion (Figure 8

).^110^,^111^ Similar findings have been reported from Amazonia, India, the Mekong, Indonesia, and the Solomon Islands.^47^,^11^–^120^ A review of submicroscopic infections detected in 31 cross-sectional surveys from 12 countries revealed that on average 69.5% of all *P. vivax* blood-stage infections were submicroscopic, and in a subset of surveys, 89–100% of submicroscopic infections were asymptomatic.^46^ The most common type of *P. vivax* infection in many areas is, therefore, both submicroscopic and asymptomatic.

**Figure 9.**
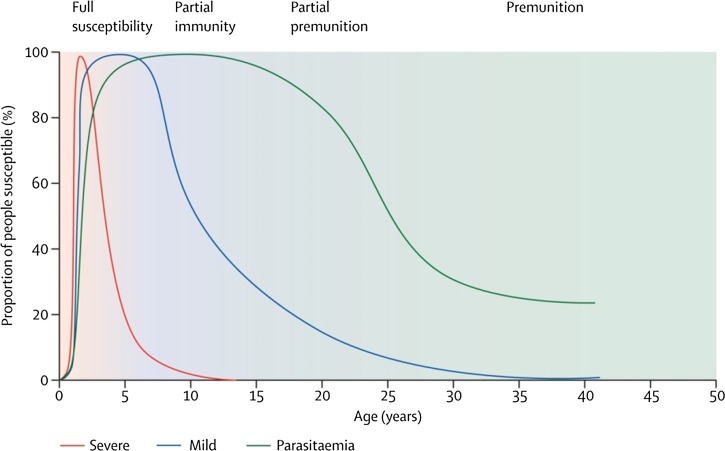
Relation between age and malaria severity in an area of moderate *Plasmodium falciparum* transmission intensity. With repeated exposure, protection is acquired first against severe malaria, then against illness with malaria, and much more slowly, against microscopy-detected parasitemia. Figure reproduced with permission from White and others.^193^

There is no known sterilizing natural immunity to malaria. People with chronic exposure appear to develop a partial immunity (parasitemia without illness) as a function of both age and the intensity of exposure, as discussed previously and illustrated in Figure 9

. The proportion of asymptomatic infections may vary considerably in endemic situations. People of all ages, and of very different previous exposure patterns to malaria, have been found to harbor asymptomatic parasitemia. This may be due to parasite and host factors other than host immunity.^121^ The protective natural immunity to infection by both *P. vivax* and *P. falciparum* in very low-transmission settings is not understood in the context of current thinking on how natural immunity is acquired.^122^

Assessing the contribution of the asymptomatic reservoir to onward transmission has proved challenging, not least due to the methodological difficulties involved. However, Barbosa and others reported gametocytemia in > 90% of individuals with asymptomatic or sub-patent *P. vivax* in Brazil^123^; and a study in Colombia including both natural and experimental infections (total patients *N* = 46) used membrane feeding assays to quantify infectivity and concluded that asymptomatic carriers were at least as infective as symptomatic patients, and also presented with the highest proportions of infectious mature gametocytes.^124^ The association between asymptomatic infection and gametocyte density is essential to understanding transmission dynamics. *Plasmodium vivax* gametocytes mature faster than *P. falciparum* gametocytes, and may, therefore, be transmitted earlier in the course of infection.^23^ Mosquito *P. vivax* infections occur at lower gametocyte densities than *P. falciparum,* and therefore, a greater proportion of transmission could occur from those with undetectable gametocytemia.^125^,^126^

The proportion of asymptomatic infections varies with transmission intensity such that in low-transmission settings, there is a higher proportion of asymptomatic carriers.^46^ By nature of being asymptomatic, these individuals are less likely to be treated, and therefore, asymptomatic infections will persist longer in a population. However, with sustained detection and treatment of symptomatic cases alone, transmission will, in theory, decrease, leading to an associated decrease in population immunity and lower frequencies of asymptomatic infections.^127^ This may be true for both *P. vivax* and *P. falciparum*. Where *P. vivax* presents a greater challenge is the hypnozoite—relapses caused by this undetectable reservoir of infection may be symptomatic or asymptomatic, and are an important source of sustained transmission where control of only blood-stage infections and mosquito interventions are implemented.^128^

## Estimates of the Clinical Burden of *P. vivax* Malaria

Reliable measures of the clinical burden of *P. vivax* have been identified as a key knowledge gap.^7^,^39^,^79^ Case estimates improve treatment targeting, guide strategic planning for control, and provide a means of monitoring the impact and progress of those interventions. A few attempts have been made to quantify clinical vivax cases, with widely divergent results.

### Case estimates.

**Figure 10.**
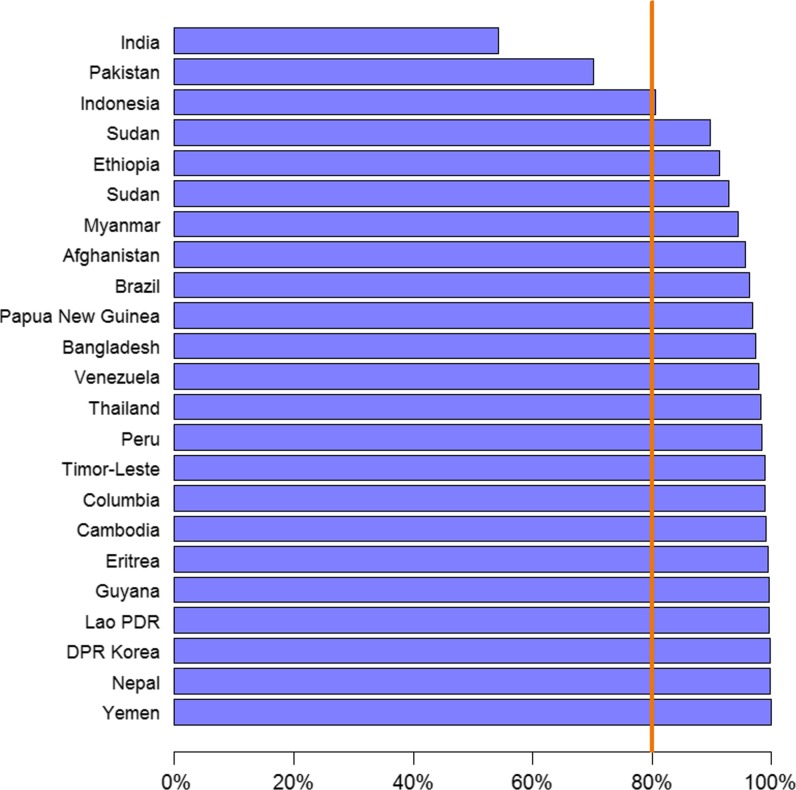
Cumulative proportion of the global estimated *Plasmodium vivax* cases accounted for by the countries with the highest number of cases. Reproduced from the World Malaria Report 2014.^132^ Lao PDR refers to Lao People's Democratic Republic and DPR Korea to Democratic People's Republic of Korea.

The most recent estimates of *P. vivax* cases, from the WHO's 2015 World Malaria Report,^36^ suggest 13.8 million (95% CI: 10.3–18.4) *P. vivax* cases in 2014. This corresponds to 6% of all malaria cases globally and 51% of all malaria cases estimated to occur outside of sub-Saharan Africa. Figure 10

shows the contribution of cases from the highest burden *P. vivax* countries in 2013. Cases from just three countries—India, Pakistan, and Indonesia—together represented > 80% of cases globally. Unlike the methodological developments estimating *P. falciparum* case numbers,^129^,^130^ limited recent efforts have been invested into *P. vivax* case estimation. Historical estimates based on pre-year 2000 data estimated a much larger case burden than the recent WHO figures (71.0–80.0 million annual cases by Mendis and others [2001] based on mid-1990s data^79^; and 132.0–391.0 million estimated by Hay and others [2004]^7^,^130^ based on a malaria endemicity map from 2002), but these cannot be considered reliable indicators of contemporary transmission. Robust methods, similar to those developed for *P. falciparum*, are required to overcome the limitations of the available data for estimating *P. vivax* cases.

### Quantifying case numbers.

In the context of varied data sources (all of them imperfect in important respects), differing methods have been used to derive case burden estimates. The WHO primarily follows a surveillance-based approach using health facility records reported to each country's Ministry of Health.^131^ The accuracy of reported cases is influenced by reporting completeness, treatment-seeking behavior, and the likelihood that a case of malaria is found to be parasite positive, all of which are adjusted for using country-specific parameters. However, for countries with less reliable routine surveillance data, model-based methods that link measures of malaria transmission with case incidence are used instead. The WHO's approach, therefore, strives to cope with the variability in diverse sources and variable quality of reported data.^131^,^132^

**Figure 11.**
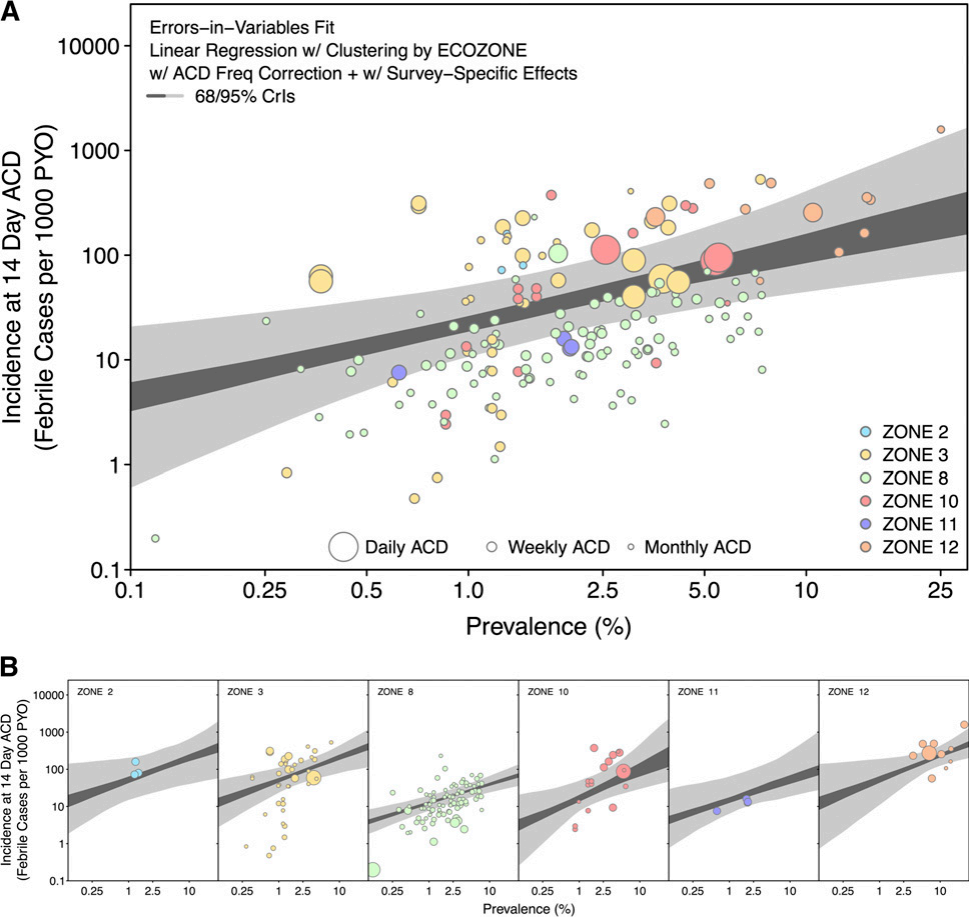
Modeled relationship between parasite prevalence and clinical case incidence for *Plasmodium vivax*. (**A**) The pooled prevalence–incidence relationship as point-wise 68% and 95% credible intervals (CrIs) based on data from all zones (Figure 5). To produce a pooled fit, the posterior of each zone was weighted by the number of observations from that zone. (**B**) The zone-specific prevalence–incidence relationships. Zone 2 is Central America, zone 3 is South America, zone 8 is Monsoon Asia (India), zone 10 is southeast Asia, zone 11 is northern Asia and Europe, and Zone 12 is Melanesia. The 95% CrIs are shown in light grey and the 68% CrIs in dark grey. The colors of the zones correspond to those shown in Figure 6B. Reproduced from Battle and others.^135^

Other epidemiologists have investigated alternative approaches based on a widely available and easily obtained epidemiological metric, the prevalence of infection (or PR). This represents the proportion of individuals infected (confirmed by microscopy or RDT) in an area at a fixed time point as measured by cross-sectional surveys.^133^ This metric was used to map the global prevalence of *P. vivax* endemicity (Figure 1).^2^ Case estimates can then be derived from the prevalence map by using a model of the relationship between prevalence of infection and clinical incidence.^129^,^130^,^134^–^136^ Figure 11

represents such a model developed for *P. vivax* that will be integrated into a cartographic approach to derive estimates of *P. vivax* clinical cases.^135^,^137^ This approach quantifies the degree of uncertainty in the predictions, highlighting regions in need of improved surveillance. Preliminary work by the authors shows that these results are of the same order of magnitude as the estimates provided by the WHO using routine surveillance data.

These alternative approaches to case estimating provide complementary and useful comparisons in continuing to leverage much-needed improved *P. vivax* surveillance and reporting.

### Trends of *P. falciparum* and *P. vivax* case count ratios.

It has been noted during successful control campaigns that *P. falciparum* is often the first species to show a decline in incidence, with *P. vivax* generally being slower to respond. In addition to relapse, this is also likely due to 1) the longer extrinsic incubation period of *P. falciparum* compared with *P. vivax*, meaning that transmission of the former is more susceptible to interventions such as insecticide-treated bed nets and indoor residual spraying which shorten the average lifespan of the vector population;^138^ and 2) the fact that infectious gametocytes appear early in a blood-stage infection of *P. vivax*, almost simultaneously with asexual parasites, in contrast to *P. falciparum* in which mature infective gametocytes take 10 days to appear in the peripheral blood after asexual parasite patency.^79^ This means that when a *P. vivax*-infected patient presents for diagnosis and treatment, onward transmission of the parasite could have already occurred and the early diagnosis and treatment strategies that are highly effective against *P. falciparum* may not be so for *P. vivax*.^79^

**Figure 12.**
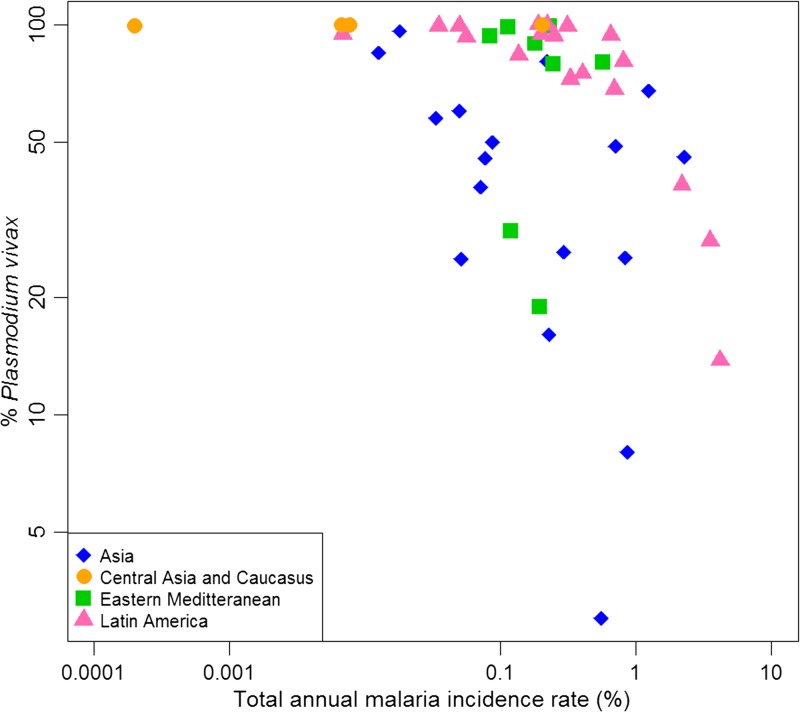
The variation of the proportion of malaria cases due to *Plasmodium vivax* with the annual malaria incidence rates in endemic countries, as published in 2001 (the rest being mainly due to *Plasmodium falciparum*) shown on a logarithmic scale. The data points are color coded and shaped by region. Asia includes Bangladesh, Bhutan, Cambodia, China, Lao People's Democratic Republic, Malaysia, Myanmar, Nepal, Papua New Guinea, Philippines, Solomon Islands, Sri Lanka, Thailand, Vanuatu, and Vietnam. Central Asia and Caucasus includes Armenia, Azerbaijan, Tajikistan, and Turkey. Eastern Mediterranean refers to Afghanistan, Iran, Iraq, Oman, Pakistan, Saudi Arabia, Syria, and Yemen. Latin America includes Argentina, Belize, Bolivia, Brazil, Colombia, Costa Rica, Dominican Republic, Ecuador, El Salvador, Guatemala, French Guyana, Guyana, Haiti, Honduras, Mexico, Nicaragua, Panama, Peru, Suriname, and Venezuela. The percentage of *P. vivax* for each country is as cases reported by the countries to World Health Organization. Note that the figure excludes data from the African region because high prevalence of the Duffy negativity phenotype results in very low *P. vivax* transmission^28^ and occasional case reports of *P. vivax* are vastly outnumbered by *P. falciparum* cases. Figure modified from the original published version,^79^ and shared by Kamini Mendis.

The phenomenon of the proportion of cases due to *P. vivax* increasing as overall cases decrease has been observed in a variety of endemic settings.^71^ Observations from Thailand,^139^ Sri Lanka,^140^ and Brazil^141^,^142^ showed the proportion of cases due to *P. vivax* greatly increased as control efforts were scaled up over time. This pattern is not always obvious from national-level data, however, because control programs are not focused uniformly across a country. Areas with the highest transmission intensity (often with more *P. falciparum*) may have fewer services available and *P. vivax*, therefore, appears to decrease more quickly as control is applied to lower transmission areas. Nonetheless, the pattern of an increasing proportion of cases due to *P. vivax* in elimination settings has also been observed in a variety of settings.^47^,^71^,^140^,^143^ All countries in the WHO 2013 World Malaria Report that reported microscopically confirmed cases found a higher proportion of *P. falciparum* in years with higher case numbers than in years with fewer.^143^ In addition, *P. vivax* was the predominant species in countries in pre-elimination and elimination phases which have a low total annual malaria incidence rate (Figure 12

).^144^,^145^

In general, decreases in the incidence of *P. falciparum* cases have been larger than those of *P. vivax*.^143^ Biological characteristics unique to *P. vivax* cause existing control strategies, particularly treatment options, to be less suitable in the final, predominantly *P. vivax*, elimination stages.^71^ The consistency of this phenomenon serves to emphasize the programmatic significance of the biological distinctions between *P. vivax* and *P. falciparum*, with malaria control having been historically aimed principally at *P. falciparum* without regard to the singular biology of *P. vivax*. Programs aiming for more rapid and thorough elimination of malaria transmission should adapt their strategies and interventions toward effectiveness against *P. vivax*; without these, standard interventions will have to be sustained over much longer periods to drive out *P. vivax* transmission.^25^,^143^,^146^

## Severe and Fatal *P. vivax* Malaria

The last decade saw a significant shift in knowledge, attitudes, and practices toward *P. vivax*. Having long been characterized as the “benign tertian” malaria,^6^,^7^,^147^–^149^ recent evidence demonstrates that a diagnosis of *P. vivax* infection can be associated with severe and fatal illness.^5^,^8^–^20^ In consequence, the species begins to become acknowledged as inherently pernicious.

### The nature of severe and fatal vivax disease.

In the absence of a species-specific case description for severe *P. vivax*,^150^ the WHO case definition for severe *P. falciparum* malaria (excluding parasite counts) has been widely applied for categorizing uncomplicated versus severe disease in patients diagnosed with *P. vivax* infection. The spectrum of clinical syndromes for both species is essentially similar.^8^ Studies of vivax morbidity and mortality from across the vivax-endemic world have reported the full spectrum of pathologies commonly attributed exclusively to *P. falciparum*, including both sequestration-related and nonsequestration-related complications.^13^–^16^,^149^,^151^–^153^ A diagnosis of acute severe vivax malaria, therefore, includes one or more of the following (numerous case reports of each exist in the literature)^8^,^10^,^13^,^19^,^149^,^154^,^155^: 1) neurological conditions, notably coma or repeated general seizures and altered consciousness; 2) hematological conditions, particularly severe anemia (< 5 mg Hb/dL), severe thrombocytopenia (< 50,000 platelets/μL), and hemoglobinuria; 3) systemic symptoms such as circulatory collapse or shock; and 4) vital organ failure, including respiratory dysfunction and acute respiratory distress syndrome, acute renal failure, splenic rupture, hepatic dysfunction, and jaundice.

The preponderance of each syndrome among severely ill patients with *P. vivax* or *P. falciparum* infection differs, and risk of death differs greatly between syndromes.^20^ For instance, metabolic acidosis, coma, and other neurological complications occur less frequently in severe vivax malaria, whereas severe anemia and acute lung injury are more commonly reported manifestations of severe *P. vivax*.^8^,^14^ Respiratory distress was associated with 15 of 17 *P. vivax* mortalities in an autopsy series in Brazil;^14^ and in Indonesian Papua, severe anemia dominated as the cause of illness among inpatients. When severe anemia occurred with respiratory distress, death became a likely outcome (OR: 65, *P* < 0.0001).^20^ Despite lower peripheral blood-stage parasitemia, the degree of anemia in severe *P. vivax* and *P. falciparum* infections is often similar.^11^

A recent global meta-analysis of 77 studies comprising 46,411 severe vivax patients revealed marked geographic patterns in the prevalence of particular clinical manifestations of severe vivax.^153^ Noteworthy was the apparently much lower incidence of severe disease in certain regions, including the Greater Mekong, than others such as India, where nearly half of the severe vivax studies were conducted. The broad pattern identified was that in high-transmission areas (such as New Guinea), severe anemia was the predominant symptom and found mainly in young infants.^12^,^76^ In lower endemicity areas (such as parts of the Americas), where adults were more likely to be affected, patients presented with a variety of vital organ dysfunctions.^153^ Strain-dependent virulence in *P. vivax* is well known and far more striking than in *P. falciparum*.^5^

### Evidence of the risks of severe and fatal *P. vivax*.

The risk of severe disease and case fatality rates (CFRs) are not firmly established for *P. vivax*. Although the WHO severe *P. falciparum* criteria apply to characterizing severe vivax, formally defining specific criteria and thresholds remains necessary to ensure consistency between studies and enable meta-analyses of the prevalence of severe disease and risks of morbidity from different manifestations of disease to be more robustly assessed.^153^ The risk of a *P. vivax* infection presenting severe symptoms is difficult to quantify because of the inherent difficulty in estimating the number of *P. vivax* infections in a community (the denominator of this risk estimate). Recent studies aiming to quantify rates of severe disease and fatality used varying metrics to represent “total *P. vivax* cases,” including hospital cases,^20^ community surveyed infections,^156^ and regional case notifications.^19^,^157^ The discrepancies in the number of cases captured through community surveys and those reporting to hospital make comparing rates of severe disease and death between studies difficult.

The risk of fatality among hospitalized *P. vivax* cases was summarized in 16 studies by Baird,^5^ of which 10 were retrospective and six prospective. Using these data and an additional 27 studies with documented species-specific numbers of severe disease and death, the median CFR among inpatients with severe disease was found to be 3.1% (interquartile range [IQR]: 0.0–9.3%).^158^ Five of these studies were able to rule out the possibility of a mixed infection with *P. falciparum* using PCR. The median CFR from severe *P. falciparum* disease where it occurred and was reported among 22 of the same hospitals was 11.6% (IQR: 4.9–22.8%). The odds of an inpatient dying from a *P. vivax* malaria infection were just under two-thirds that of those with severe *P. falciparum* infections (OR: 0.63, 95% CI: 0.52–0.77). This ratio is unlikely to reflect the relative risks of dying among patients who acquire malaria in the community. Nonetheless, the studies do show that severe cases and deaths due to *P. vivax* can occur in all endemic regions.

Population-based risks of death have rarely been compared between parasites. Where they have, the risk with *P. vivax* was less than half that associated with *P. falciparum*.^20^ This large prospective population-based study (*N* = 45,525 clinical *P. vivax* cases) estimated the risk of severe disease to be one in 270 clinical episodes, and death was one in 3,959. The equivalent risks among 72,721 clinical episodes of *P. falciparum* were one in 185 and one in 1,742, respectively, illustrating a significantly different risk of death between species. Nonetheless, this study and many others firmly support abandoning the dogma that *P. vivax* is an intrinsically benign and generally harmless species.

### Comorbidities and malnutrition.

Although it is apparent that *P. vivax* mono-infections can be associated with severe and fatal illness,^5^,^14^,^151^ coinfections with other pathogens may be playing important roles influencing the likelihood of those poor outcomes, as reported with *P. falciparum*.^159^–^161^ The Brazilian autopsy study previously mentioned^14^ identified comorbidities as important in 13 of the 17 deaths considered. Similarly, a series of Brazilian intensive care patients reported over half of those admitted with a primary diagnosis of *P. vivax* malaria, (14/24) suffered at least one acute or chronic comorbidity.^16^

Few studies have described comorbidities with *P. vivax*. Understanding the impact of coinfections on clinical outcome is nonetheless vital to assuring appropriate treatment courses and case management, as has been demonstrated with *P. falciparum*.^159^ For instance, dengue and *P. vivax* show spatial overlap in distributions^2^,^162^,^163^ and their coinfection presents significant diagnostic and treatment challenges.^164^–^168^ Another important geographically overlapping family of pathogens is the soil-transmitted helminths (STHs) that also cause anemia,^165^–^169^ but only a handful of studies have examined their association with *P. vivax*. A study of Brazilian schoolchildren found STHs had a protective effect against severe anemia during *P. vivax* infections,^170^ whereas a study of pregnant women on the Thai–Myanmar border reported a reduced risk of clinical vivax cases (AOR: 0.29, 95% CI: 0.11–0.79) in women infected by *Ascaris lumbricoides*, but not with other STH species.^171^ Further work is needed to better understand these apparently complex interactions and how control and treatment should be adapted to account for the coinfection. As highlighted by Boel and others, a potentially negative impact of mass deworming programs on the pathogenesis of *P. vivax* disease severity complicates and leaves unclear the use of control interventions without fully understanding the interactions between coinfections.^171^

Malnutrition is also an important predisposing factor for severe outcomes of *P. vivax* infection and is commonly diagnosed in severe vivax malaria patients globally.^13^,^16^,^155^,^172^ Given the high prevalence of malnutrition across malaria-endemic zones, further studies are needed to better establish the risks of *P. vivax* disease associated with malnutrition, and the programmatic possibilities for interventions addressing these simultaneously.

## Epidemiology of Chloroquine-Resistant *P. vivax*

Chloroquine has been the frontline drug for treating asexual blood-stage *P. vivax* parasites during acute clinical attacks since 1946.^53^ Administered in combination with the hypnozoitocidal drug primaquine, this drug combination provides radical cure of *P. vivax* infection by also killing the hypnozoites responsible for relapses. Despite growing evidence of chloroquine resistance across heavy-burden *P. vivax* regions, this drug combination remains the first-line treatment in many countries.^36^ Chloroquine resistance poses a major and worsening threat to the health of millions of patients. A fuller technical description of this problem may be found in the article on diagnosis and treatment of *P. vivax* by Price and others in this supplemental volume.^173^ What follows here summarizes the problem in an epidemiological context.

### Defining chloroquine resistance.

The standard chloroquine total dose of 25 mg/kg is administered as three daily doses.^174^ This typically clears chloroquine-sensitive blood-stage *P. vivax* parasites within 48 hours. The relatively slow in-host elimination of chloroquine sustains minimally effective concentrations against sensitive *P. vivax* in blood for approximately 35 days.^53^ Any recurrence with chloroquine administered at 100 nm concentration at this dosing may be classified as resistant regardless of its life-stage origin (recrudescence, relapse, or reinfection; see Figure 4).^175^ A 28-day in vivo test for chloroquine resistance has been adopted based on this rationale,^175^–^177^ and the 28-day cumulative incidence of recurrences with > 100 ng/mL chloroquine and its major metabolite, desethyl chloroquine, is the current standard metric for risk of treatment failure due to parasite resistance to chloroquine.

### The epidemiology of chloroquine resistance.

**Figure 13.**
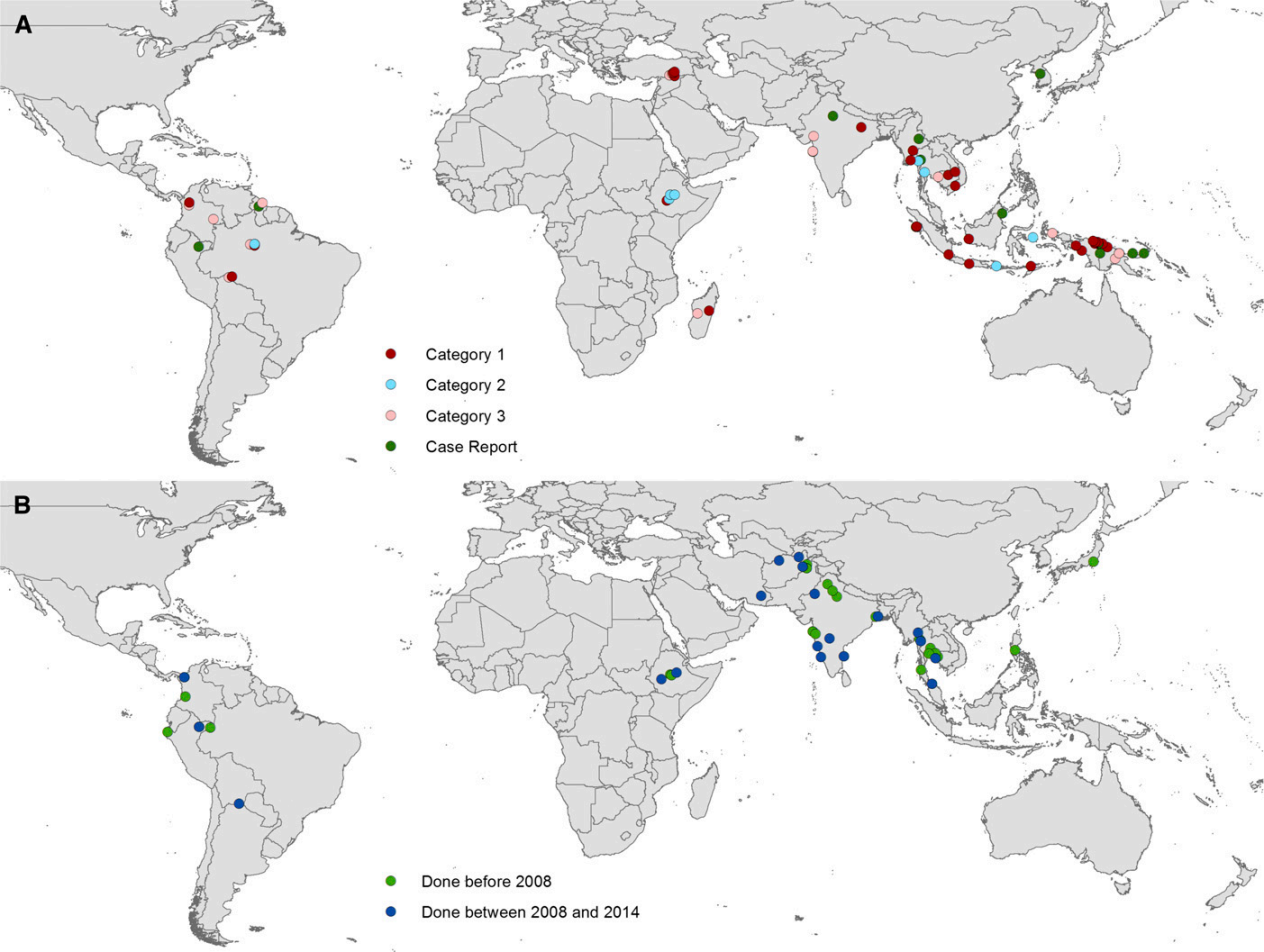
The locations of (**A**) documented chloroquine-resistant and (**B**) chloroquine-sensitive *Plasmodium vivax*. Chloroquine resistance was categorized according to the strength of evidence^182^: Category 1: > 10% recurrence by day 28, irrespective of confirmation of adequate blood chloroquine concentration; Category 2: confirmed recurrences by day 28 within reported whole-blood chloroquine concentration of > 100 nm; and Category 3: > 5% recurrences by day 28, irrespective of chloroquine concentration. Chloroquine sensitivity was confirmed if patients had enrolled after a symptomatic clinical illness, fewer than 5% recurrences had occurred by day 28, no primaquine was given before day 28, and studies had a sample size of at least 10 patients. Case reports were observations in individual patients of treatment failure during chloroquine prophylaxis, prolonged parasite clearance or *P. vivax* recurrence following treatment. Figure reproduced from Price and others (2014).^182^

The first report of *P. vivax* chloroquine resistance dates to 1989 in Australian travelers visiting Papua New Guinea.^178^ High-grade chloroquine resistance has been described in and around this area,^174^,^179^–^182^ and evidence of resistance is now reported from many high-burden countries across all three endemic continents,^182^ including the Americas.^183^ Figure 13

illustrates the findings from the relatively scant surveys reported globally up to April 2014.

The WHO have standard protocols for therapeutic efficacy surveys for the routine monitoring of drug sensitivity to inform national drug policies.^177^ Nevertheless, more detailed meta-analyses of research studies aiming to monitor the spread of resistant strains globally are currently hindered by both variability in survey design and the paucity of them.^182^ Further, ineffective chloroquine treatment because of poor-quality drugs represents a confounding factor in assessing resistance. A review of antimalarial drug quality assessments found that 27% of 2,182 chloroquine samples failed a quality test.^183^ Although many of these surveys were on drug samples from sub-Saharan Africa, Asian countries were also well represented in the review. The issue of substandard drugs adds a further dimension of complexity to monitoring chloroquine resistance in the field.

### The clinical implications of chloroquine resistance.

Ineffective therapy due to resistance causes an increased burden of disease by extending the duration of clinical symptoms, thus increasing the likelihood of onset of severe and threatening illness and the probability of onward transmission. The neglect of *P. vivax* research and development investments has left a void of *P. vivax*-focused clinical trials and thus few validated alternative treatment options. Evidence of resistance prompted shifts in antimalarial policy, driving countries to turn to new and untested drug combinations with artemisinin-combined therapies (ACT) to replace chloroquine.^185^,^186^ However, a recent review of ACT efficacy and safety from 35 studies in 13 countries across three continents reports good efficacy against asexual blood stages of *P. vivax* (except those ACTs employing pyrimethamines).^136^,^187^ Primaquine administered 25 days following dihydroartemisinin plus piperaquine was determined safe and effective as a radical cure.^136^ An important hurdle to scaling-up these new treatments and preventing ongoing circulation of ineffective treatment, however, is the greater cost associated with ACT treatment ($0.92–3.85 per adult course) over chloroquine ($0.07).^187^ See Price and others for further discussion of the clinical aspects of these issues.^173^

There is at present no conclusive evidence of *P. vivax* developing resistance against primaquine; not least because attributing treatment failure to primaquine resistance is not straightforward.^188^ This is in part due to the complex metabolism of primaquine in the human host, which directly determines the availability of drug metabolites active against the parasites. Evidence of a potentially quite common “poor metabolizer” human phenotype of CYP2D6 results in treatment failures that should not be attributed to resistance.^189^ The inability to obtain practical and reliable estimates of the prevalence and distribution of resistance to primaquine by *P. vivax* hypnozoites leaves us blind to this potentially important problem.

## Conclusions

The epidemiology of *P. vivax* cannot be conveniently defined by a simple set of characteristics and patterns. Instead, it is highly heterogeneous between settings and populations, influenced by a plethora of features including local mosquito vector species, transmission intensity, relapse behaviors, host risk factors, availability and efficacy of treatment, malnutrition and prevalence of comorbidities, to name a few. This article has attempted to draw generalities where possible, but also emphasize key variability. The consequence of this heterogeneity is that a single rule book cannot guide control programs everywhere: strategies must vary according to each area's local epidemiology, and evidence is needed from across these different settings to allow programs to target local requirements. This had been lacking, but recent years have seen a surge of interest and funding specifically focused on *P. vivax*. Studies are now being carried out across the *P. vivax*-endemic world to address the knowledge gaps referred to in this review and to allow local control strategies to be optimized. See the paper in this supplement detailing those research gaps (Bassat and others).^190^

Relapsing hypnozoites mean that significant reductions in *P. vivax* case burden may only become evident a few years after decreases of *P. falciparum*, due to the hidden reservoir of silent *P. vivax* parasitemia. Control and elimination of *P. vivax*, therefore, demands persistence and long-term commitment. Furthermore, the hidden iceberg of both submicroscopic asymptomatic parasitemia and hypnozoites make currently available tools insufficient to assess the true prevalence of infection. Improved surveillance and hospital-based studies may help to unravel the relationships between different epidemiological metrics to allow better estimates of asymptomatic infections, uncomplicated cases, severe cases, and deaths.

Treatment inadequacies severely hinder *P. vivax* control. Continued treatment with chloroquine in areas of known resistance puts millions of patients at risk, whereas radical cure with primaquine is not widely used due to the rational fear of harm to G6PD-deficient patients.^70^,^125^ Widespread safe access to radical cure would help overcome the resilience of endemic *P. vivax* to control efforts and could substantially impact the parasite reservoir.

A clearer picture of *P. vivax* malaria as a widespread and potentially severe and fatal infection is emerging. We may confidently state that infection by *P. vivax* is certainly not universally “benign.” Recognition of the potential for *P. vivax* to cause severe disease or mortality, combined with the sheer scale of the PAR of infection (35% of the global population) and the imperfect toolkit available to fight *P. vivax*, begin to define a very serious clinical and public health problem in urgent need of greater attention.

## Figures and Tables

**Table 1:** Areas and populations at risk* of *Plasmodium vivax* malaria in 2010 by World Health Organization region^2^

	Area (million km^2^)			Population (million)		
	Unstable	Stable	Any risk	Unstable	Stable	Any risk
African region	17.36	1.47	18.83	19.23	36.80	56.02
Region of the Americas	1.38	8.08	9.46	87.66	49.79	137.45
Eastern Mediterranean region	4.24	0.60	4.84	176.11	47.37	223.48
European region	0.40	0.02	0.42	15.72	1.40	17.12
Southeast Asia region	2.11	3.88	5.99	691.71	748.38	1,440.09
Western Pacific region	3.06	1.30	4.36	533.04	81.16	614.20
World	28.55	15.35	43.90	1,523.47	964.90	2,488.37

* Risk is stratified into unstable risk (*Plasmodium vivax* annual parasite incidence [*Pv*API] < 0.1 per 1,000 people per annum) and stable risk (*Pv*API ≥ 0.1 per 1,000 people per annum).
